# The Prevalence of the McKenzie Nerve in Cadavers: Its Clinical Application to Intradural Rhizotomy for Spasmodic Torticollis

**DOI:** 10.7759/cureus.95689

**Published:** 2025-10-29

**Authors:** Priyanka N Sharma, Manoj M Kulkarni, Achleshwar R Gandotra

**Affiliations:** 1 Anatomy, Smt. B. K. Shah Medical Institute and Research Centre, Sumandeep Vidyapeeth (Deemed to be University), Vadodara, IND

**Keywords:** c1 nerve root, cervical-spine, mckenzie nerve, spasmodic torticollis, spinal accessory nerve, spinal cord, ventral root

## Abstract

Introduction

Spasmodic torticollis may require surgical intervention, such as intradural rhizotomy, when medical treatments are ineffective. It is important to recognize the nerve of McKenzie, which is a connection between the C1 ventral root and the spinal accessory nerve. It exists in some cases, and if overlooked, it can lead to persistent symptoms. The findings establish a foundation for enhancing outcomes in neurosurgical interventions for spasmodic torticollis by improving understanding of this uncommon neural entity.

Methods

We included 30 formalin-fixed adult human cadavers (15 female and 15 male) without any previous spinal surgical procedures, pathology of the spinal cord, or non-traumatic pathology. The age range of the cadavers was 60 to 95 years (mean: 77.5 years). Utilizing a posterolateral approach, the craniocervical junction was exposed. The occipital bone and posterior neural arch of the C1-C7 vertebrae were excised, and the meninges were cleaned. The spinal accessory nerve course was traced, and the McKenzie nerve and surrounding vascular relations were identified and documented.

Results

The McKenzie nerve was observed in six out of 60 sides (10% prevalence). Prevalence was equal between sexes but more frequent on the right side (66.7%). Mean nerve length was greater on the right (5.8 ± 0.61 mm) compared to the left side (5.05 ± 0.07 mm). Bilateral occurrence was noted in two specimens. Two specimens exhibited bilateral absence of C1 dorsal roots. The presence of the McKenzie nerve was statistically significant (p=0.04).

Conclusion

In our research, we observed that the McKenzie nerve tends to be longer and more frequently found on the right side. In two specimens, it was present bilaterally, highlighting the necessity for a comprehensive bilateral examination due to the possibility of it occurring on both sides. These findings highlight the necessity for meticulous attention during surgical interventions for spasmodic dystonia. Failure to identify the McKenzie nerve could result in incomplete symptom relief. These observations may contribute to improved surgical outcomes.

## Introduction

The McKenzie nerve, an intradural communication between the spinal accessory nerve (SAN) and the ventral root of C1, holds significant importance in the surgical management of spasmodic torticollis. This idiopathic focal dystonia of the cervical musculature results in involuntary and irregular contractions, leading to abrupt cephalic movements [1-7]. Treatment of spasmodic torticollis initially includes muscle relaxants and botulinum injection, and after resistance to botulinum injections, alternative management strategies include selective peripheral denervation, the Bertrand procedure, and central procedures such as bilateral thalamotomy or bilateral globus pallidus deep brain stimulation procedures [8]. Although a majority of reports have mentioned about the McKenzie nerve during rhizotomy, a notable proportion of studies reported unsatisfactory outcomes [2,3,8-12]. Friedman et al. [2] performed selective intradural rhizotomy, in which they transected ventral C1, C2, and C3 rootlets and SAN rootlets for denervation of the sternocleidomastoid in patients with spasmodic torticollis. They also made an effort to find the McKenzie nerve, which causes strong sternocleidomastoid muscle contraction when stimulated in 50% of patients [13]. Jetjumnong et al. [6] and Aljuboori et al. [8] reported that failure to section this nerve leads to complications such as continued torticollis because the branch is thought to convey motor fibers from the accessory nerve to the first cervical nerve while minimizing the risk of swallowing difficulties and trapezius paralysis [7].

In certain cases, the presence of the McKenzie nerve, if unrecognized, may lead to persistent symptoms. The existing literature lacks comprehensive anatomical studies on the McKenzie nerve, and descriptions of surgical procedures frequently omit reference to this nerve. These observations provide a foundation for enhancing neurosurgical outcomes in the treatment of spasmodic torticollis by advancing the understanding of this rare neural structure.

## Materials and methods

The present study was conducted at the Department of Anatomy of a teaching medical institute in Gujarat, India. Ethical approval to undertake the present study was obtained from the Institutional Ethical Committee (IEC).

We included 30 formalin-fixed adult human cadavers (15 female and 15 male) without any previous spinal surgical procedures, pathology of the spinal cord, or non-traumatic pathology, encompassing 60 sides in a prone position of the head and neck region. The average age at the time of death was 77 years (range: 60-95 years). The head was stabilized using a fixation device. Through a posterolateral approach, the soft tissue overlying the craniocervical junction was removed. Using a high-speed surgical drill, the occipital bone and posterior neural arch of the cervical vertebrae from C1 to C7 were excised, and the underlying dura mater covering the craniocervical junction to the cervical spine was incised using dissecting scissors [12]. The arachnoid mater was carefully cleaned, ensuring that the denticulate ligaments remained intact. Rootlets that emerged dorsal to the denticulate ligament were classified as dorsal rootlets, whereas those ventral to it were identified as ventral rootlets. Using a surgical magnification loupe with 4x magnification, the SAN (XI) was located, and its path was followed. During this process, the communicating branch between the SAN (XI) and the ventral C1 root, known as the McKenzie nerve, was identified, along with its association with nearby vascular structures. To further verify these findings, a specimen was excised from the lower medulla to the cervical spinal cord and then mounted on a wax block for additional analysis. All observations were recorded in a standardized format, including measurements of nerve lengths, type, and positional relationships. The length of the nerve, when present, was measured with a digital vernier caliper. These data were then compiled into a comprehensive database for statistical analysis. The data were analyzed using SPSS Version 23 (IBM Corp., Armonk, NY) and using Welch’s t-test with the level of significance (p-value) set at 0.05.

## Results

**Figure 1:**
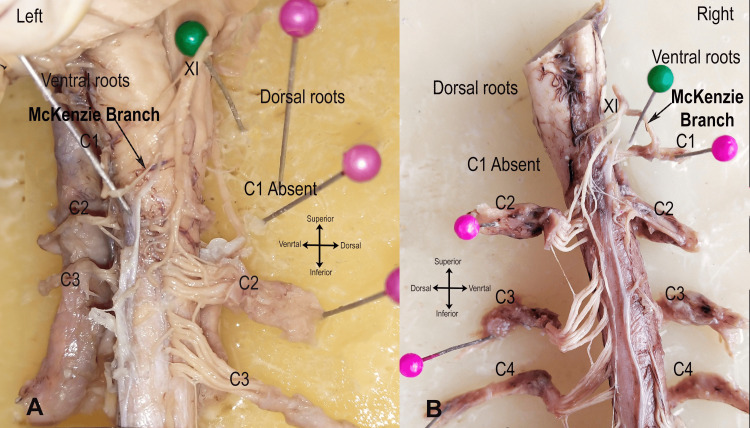
Cadaveric specimen placed on wax board exhibiting the McKenzie nerve (black arrow), with dorsal C1 roots absent on both sides. (A) Left side. (B) Right side. XI, spinal accessory nerve

The McKenzie nerve was observed in six out of 60 examined sides, indicating a prevalence of 10%. In all instances, it manifested as a single, short, and thick communicating nerve between the ventral root of C1 and the SAN (XI). The prevalence was equally distributed between males and females. However, it exhibited a higher frequency on the right side, accounting for four (66.7%) out of six cases. The mean length of the McKenzie nerve was greater on the right side (5.8 ± 0.61 mm; range 5-6.5 mm) compared to the left side (5.05 ± 0.07 mm; range 5-5.1 mm). Bilateral occurrence was noted in one male and one female specimen. In five out of six cases, the nerve was obscured anteriorly by the adventitia of the vertebral artery. The McKenzie nerve connected to the ventral root of C1 by piercing the highest denticulate ligament in four (66.7%) out of six cases. In two (33.3%) cases, the nerve did not pierce the ligament due to a higher origin from the SAN. Two specimens exhibited the absence of dorsal roots of C1 bilaterally (Figure 1). The presence of the McKenzie nerve was found to be statistically significant (p=0.04) (Table 1).

**Table 1:** Comparison of McKenzie nerve length on sides

Side	Presence of the McKenzie nerve on sides	Length of the McKenzie nerve, mean ± SD (mm)	t-Value	Df	p-Value
Right	4	5.80 ± 0.61	2.43	3.15	0.04
Left	2	5.05 ± 0.07			

## Discussion

​Comprehending the anatomy of the dorsal rootlet of the C1 nerve and its components is advantageous for neurosurgeons. For example, conducting a rhizotomy on the dorsal roots of the upper cervical nerves has demonstrated some efficacy in the treatment of facial and oropharyngeal cancer [14]. The McKenzie nerve has been implicated in some of these recalcitrant cases. Since the ventral root of a spinal nerve is expected to contain motor fibers, it is likely that the C1 nerve supplies motor fibers to the SAN, potentially aiding in the innervation of the sternocleidomastoid and trapezius muscles, alongside the rest of the SAN. Additionally, the craniocervical junction acts as a transition zone between the occipital and cervical somites and cranial nerve nuclei. Thus, this region includes a mix of tissues originating from both somatic sources and pharyngeal arches. This blend of tissue types supports the findings, both functionally and embryologically, that the fibers from the accessory ganglia of the spinal trunk of the accessory nerve run caudally to join the anterior roots of the first cervical nerves and to course peripherally with these fibers [15,16]. This study aimed to assess the prevalence and anatomical characteristics of the McKenzie nerve in cadaveric specimens, providing valuable information for neurosurgical interventions in spasmodic torticollis because it also supplies the muscles which are supplied by SAN. The McKenzie nerve was observed in six (10%) specimens examined, with equal distribution between sexes but more frequent occurrence on the right side. McKenzie [13] found the McKenzie nerve in 50% patients, Tubbs et al. [14] found it in 70% specimens, both of which were reported as a substantially higher prevalence [17], Friedman et al. [2] found it in 3.4% patients, and Saylam et al. [18] and Oh et al. [15] found it in 4.1% and 7% of specimens, respectively (Table 2). The relatively low prevalence underscores the potential for this nerve to be overlooked during surgery, possibly resulting in persistent symptoms in some patients. Our findings regarding the anatomical characteristics of the McKenzie nerve corroborate previous descriptions. We observed it as a single, short, thick communicating nerve between the ventral root of C1 and the SAN, consistent with Oh et al.'s [15] description of the "spinal type" McKenzie nerve. The mean length measured (5.05-5.8 mm) is comparable to that reported by Tubbs et al. [12] (5.2-6 mm). The McKenzie nerve's close proximity to the vertebral artery's adventitia, as observed in our study and others [12,19], emphasizes the necessity for extreme caution during surgical procedures. This proximity to critical vascular structures increases the potential for complications if the nerve is not accurately identified and managed. We found that the McKenzie nerve pierced the first denticulate ligament in 66.3% of cases where it was present, a higher percentage than the 52.4% reported by Tubbs et al. [12]. This anatomical relationship is crucial for surgeons to comprehend, as the nerve may be concealed within the ligament, presenting challenges in identification. Moreover, in cases of severe mechanical stress or pre-existing conditions such as Chiari malformation, a taut denticulate ligament could potentially increase stress on the nerve and spinal cord [20]. The McKenzie nerve was frequently found adjacent to the vertebral artery’s adventitia. Variations such as transverse foramen could potentially alter the pathway or position of the vertebral artery, increasing the risk of vascular injury during surgical procedures at the craniocervical junction.

**Table 2:** McKenzie nerve prevalence comparison with other studies

Authors	Sample size	Prevalence of McKenzie nerve	Bilateral McKenzie nerve present
McKenzie et al. (1955) [13]	10 patients	5 (50%)	-
Friedman et al. (1993) [2]	58 patients	2 (3.4%)	-
Oh et al. (2003) [15]	100 cadaveric specimens	7 (7%)	1
Saylam et al. (2009) [18]	49 cadaveric specimens	2 (4.1%)	1
Tubbs et al. (2014) [12]	30 cadaveric specimens	21 (70%)	4
Present study	60 cadaveric specimens	6 (10%)	2

The bilateral occurrence of the McKenzie nerve in two specimens (one male, one female) in our study is noteworthy. This finding underscores the importance of thorough examination on both sides during surgical interventions. The clinical significance of the McKenzie nerve in spasmodic torticollis has been demonstrated in several studies. Friedman et al. [2] found that severing this nerve during rhizotomy procedures led to more effective symptom relief compared to other approaches. Recent clinical studies have confirmed the nerve's role through electrostimulation, which induced strong contractions of the sternocleidomastoid muscle [2,6,8]. These findings, combined with our anatomical observations, emphasize the importance of identifying and carefully managing the McKenzie nerve during surgical interventions for spasmodic torticollis. Failure to recognize and address this nerve when present may result in incomplete symptom resolution. While this study provides valuable insights, it is limited by its reliance on cadaveric specimens, which may not fully represent the variability in living patients. Future research could benefit from combining anatomical studies with intraoperative observations with a large sample size and long-term clinical outcomes to further elucidate the role of the McKenzie nerve in spasmodic torticollis.

## Conclusions

In our research, we observed that the McKenzie nerve tends to be longer and more frequently found on the right side. In two specimens, it was present bilaterally, highlighting the necessity for a comprehensive bilateral examination due to the possibility of it occurring on both sides. These findings highlight the necessity for meticulous attention during surgical interventions for spasmodic dystonia. Failure to identify the McKenzie nerve could result in incomplete symptom relief. These observations may contribute to improved surgical outcomes.

## References

[1] Martin PR (1982). Spasmodic torticollis: a behavioral perspective. J Behav Med.

[2] Friedman AH, Nashold BS Jr, Sharp R, Caputi F, Arruda J (1993). Treatment of spasmodic torticollis with intradural selective rhizotomies. J Neurosurg.

[3] Fouad W (2011). Surgical management of spasmodic torticollis. Alexandria J Med.

[4] Wang J, Li J, Han L (2015). Selective peripheral denervation for the treatment of spasmodic torticollis: long-term follow-up results from 648 patients. Acta Neurochir (Wien).

[5] Zhang S, Zeng N, Wu S, Wu HH, Kong MW (2024). Research progress in spasmodic torticollis rehabilitation treatment. World J Clin Cases.

[6] Jetjumnong C, Norasetthada T (2022). Modified McKenzie-Dandy operation for a cervical dystonia patient who failed selective peripheral denervation: a case report and literature review. Surg Neurol Int.

[7] Ravindran K, Ganesh Kumar N, Englot DJ, Wilson TJ, Zuckerman SL (2019). Deep brain stimulation versus peripheral denervation for cervical dystonia: a systematic review and meta-analysis. World Neurosurg.

[8] Aljuboori Z, Ball T, Nauta H (2020). Modified McKenzie procedure for the treatment of fixed painful torticollis. Neurosurg Focus Video.

[9] Dandy WE (1930). An operation for the treatment of spasmodic torticollis. Arch Surg.

[10] Gündüz A, Korkmaz B, Kiziltan ME (2014). Effective treatment of congenital muscular torticollis using botulinum toxin. J Craniofac Surg.

[11] Fabinyi G, Dutton J (1980). The surgical treatment of spasmodic torticollis. Aust N Z J Surg.

[12] Tubbs RS, Benninger B, Loukas M, Cohen-Gadol AA (2014). The nerve of McKenzie: anatomic study with application to intradural rhizotomy for spasmodic torticollis. Br J Neurosurg.

[13] McKenzie KG (1955). The surgical treatment of spasmodic torticollis. Clin Neurosurg.

[14] Tubbs RS, Loukas M, Yalçin B, Shoja MM, Cohen-Gadol AA (2009). Classification and clinical anatomy of the first spinal nerve: surgical implications. J Neurosurg Spine.

[15] Oh CS, Chung IH, Lee KS (2003). Topographical anatomy on the communicating branch between the spinal accessory nerve and the anterior root of the first cervical nerve. Surg Radiol Anat.

[16] McKenzie KG (1924). Intrameningeal division of the spinal accessory and roots of the upper cervical nerves for the treatment of spasmodic torticollis. Surg Gynecol Obstet.

[17] Carsky K, Iwanaga J, Clark M, Aysenne A, Tubbs RS (2021). A nerve of McKenzie with a variant communicating branch between the vagus nerve and cranial root of the accessory nerve: a cadaveric case report. Cureus.

[18] Saylam CY, Orhan M, Ikiz Z, Uçerler H, Zileli M (2009). The incidence and anatomical features of the McKenzie branch: a cadaver study. Turk Neurosurg.

[19] Ogut E, Guzelad O, Yıldırım FB (2013). Investigation of accessory transverse foramen in dry cervical vertebrae: incidence, variations, types, locations, and diagnostic implications. Egypt J Forensic Sci.

[20] Ceylan D, Tatarlı N, Abdullaev T (2012). The denticulate ligament: anatomical properties, functional and clinical significance. Acta Neurochir (Wien).

